# A translational perspective of the malignant hematopoietic proteoglycome

**DOI:** 10.1186/s13578-025-01360-7

**Published:** 2025-02-20

**Authors:** Naomi Borghini, Mirca Lazzaretti, Paolo Lunghi, Giorgio Malpeli, Stefano Barbi, Roberto Perris

**Affiliations:** 1 COMT– Centre for Molecular and Translational Oncology, University of Parma, Parco Area delle Scienze, 11/A, Parma, 43124 Italy; 2 Department of Chemical and Life Sciences and Environmental Sustainability, University of Parma, Parco Area delle Scienze, 11/A, Parma, 43124 Italy; 3 Department of Life Science, Health, and Health Professions, Link Campus University, Via del Casale di San Pio V, 44, Roma, 00165 Italy; 4 Department of Diagnostics and Public Health, University and Hospital Trust of Verona, Piazzale L.A. Scuro, 10, Verona, 37134 Italy

**Keywords:** Proteoglycans, Leukemia, Lymphoma, Hematopoiesis, Diagnostics, Biomarker, Drug resistance, Immunotherapy

## Abstract

Proteoglycans are an ample family of complex extracellular matrix/cell surface components known to impact on virtually all biological processes that take place during life of a human being, in its healthy and diseased conditions. They are consolidated multivalent regulators of the behaviour of normal and malignant hematopoietic cells because of being critical components of their membranes, because of their pivotal role as multifaceted factors of the hematopoietic niches and because of acting as pillars of the tumour microenvironment. Likewise, they act as promoters of the growth, spreading and therapeutic resistance of diseased hematopoietic cells, also by modulating intracellular processes through a dual utilization of core protein domains and their glycosaminoglycan side chains. The intricate pattern of expression of the myriads of proteoglycan isoforms generated by differential glycanations of the core proteins is differentiation- and cell activation-dependent and often associates with genomic aberrations and gene amplifications. Selected proteoglycans stand out as widely recognized, disease type-specific markers and as alluring but still unappreciated therapeutic targets. We therefore pose here a clinical-translational view on the hematopoietic *proteoglycome* to highlight its underestimated biological and pathological significance during normal and neoplastic hematopoiesis. We underscore the potential of several proteoglycans to be exploited as key markers for prognostication and therapeutic targeting of hematopoietic cancers.

Proteoglycans are prospected to represent the most diversified family of proteins of the human proteome and strongly contribute to the diversification of the human glycome (Fig 1). The family was originally defined to be composed of proteins possessing the unique trait of being glycanated with glycosaminoglycan (GAG) chains [1, 2]. However, this classification criterium was later surpassed by the nucleotide sequence homology criterium conventionally used for delineating protein families of vertebrate genomes. The most recently proposed classification of proteoglycans [3] enforces the numerosity and multifunctional nature of the macromolecules and consolidates their subgrouping according to the cellular/subcellular localization, overall gene/protein homology and the presence of discrete structural-functional modules within their protein cores. Consequently, proteoglycans are divided in four categories, including intracellular, cell membrane-associated, pericellular and extracellular ones [3], with the predominant portion being ECM components fundamental for assembly and remodelling of matrices throughout the body.

**Fig. 1:**
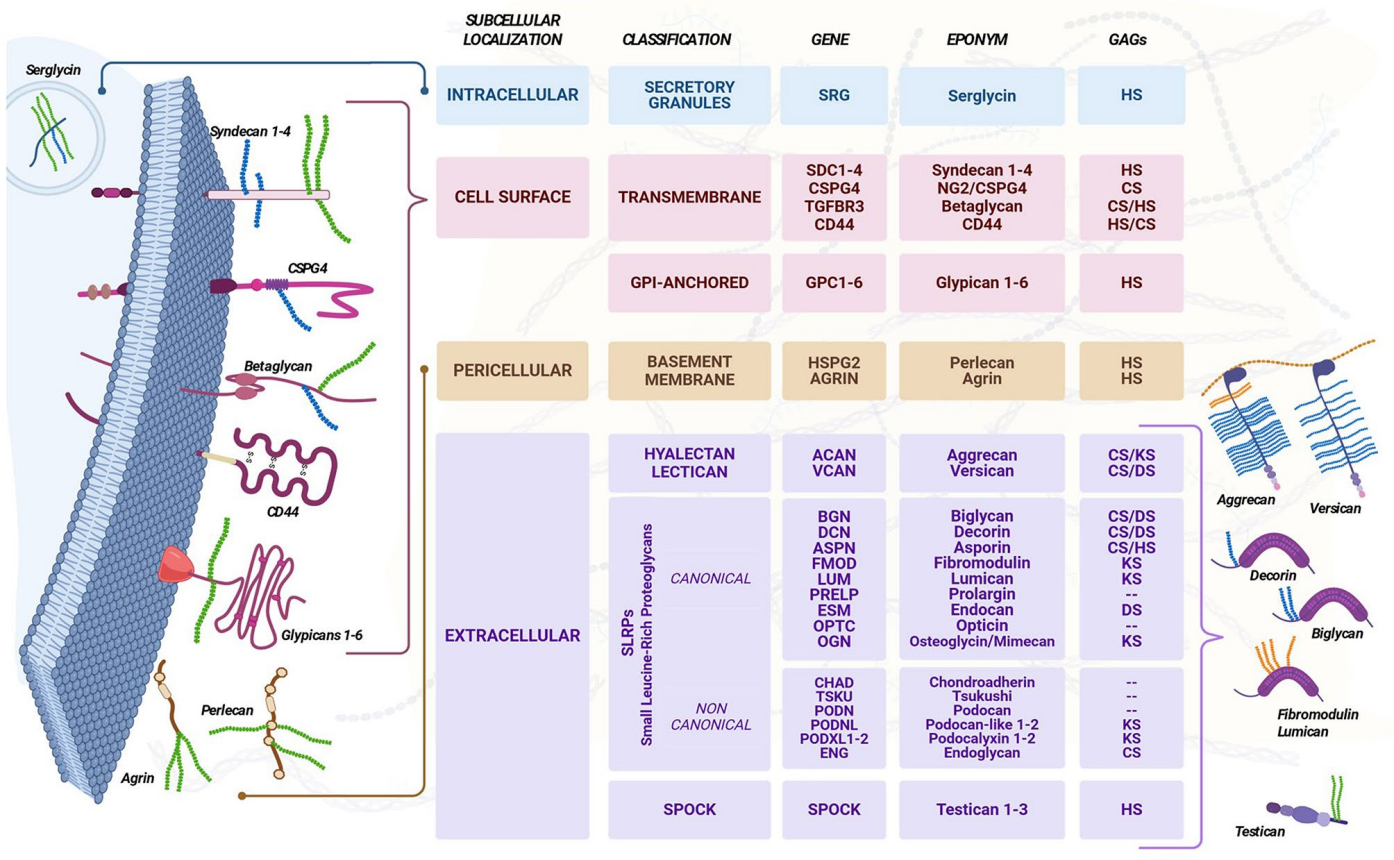
Comprehensive classification of proteoglycans. GAG, Glycosaminoglycan; HS, Heparan Sulfate; CS, Chondroitin Sulfate; KS, Keratan Sulfate; DS, Dermatan Sulfate

Proteoglycans collectively show a ubiquitous tissue and cell phenotypic distribution and a critical involvement in virtually all known biological processes [4, 5]. Hence, mutations, gene deletions, aberrant expression and post-translational modification of proteoglycans have been implicated in numerous disease conditions including cancer [6–12]. In cancer cells, upregulation, or de novo expression, of proteoglycans confers malignancy due to enhancement of the responsiveness to growth-promoting signals, sustained survival and potentiated dissemination and invasive capabilities. Meanwhile, changes in the proteoglycan arrangement in the ECM surrounding cancer cells renders their microenvironment more favourable for the maintenance of a latent, quiescent state, as well as shapes a highly dynamic milieu for the transmission of activating signals promoting their propagation and spreading. In the present review we discuss the biological significance of proteoglycans in healthy and neoplastic hematopoietic cells and the potential clinical impact of the aberrant proteoglycan patterns that can be disclosed in hematopoietic malignancies. We further address the pathological implication of these patterns and how unique variations of the hematopoietic *proteoglycome* could effectively be exploited in the treatment of cancer patients.

## Constructing the hematopoietic stem cell niche with proteoglycans

In the healthy bone-marrow, proteoglycans are key components of the hematopoietic stem cell niches, being critically involved in the preservation of the homeostatic balance of the bone-marrow microenvironment, while more discretely controlling the dynamics of stem/progenitor cell development. Prevalent proteoglycans of the bone-marrow stromal ECM include, *Perlecan*, *Agrin*, *Decorin*, *Biglycan* and *Versican*, with the preponderance of these proteoglycans being jointly contributed by the stromal and vascular cells [13–17]. However, a finer (single-cell) expression mapping involving the specialized cells (i.e., CAR and Canopy cells) of the periosteal stem cell niche remains to be accomplished. *Syndecans-1/CD138*,* -2 (CD362)*, *-3* and *− 4*, *Glypican-1* and − *4* and *NG2/CSPG4* are the primary cell surface-bound proteoglycans that have been found to be associated with the surfaces of the different stem cell subpopulations and bone-marrow stromal cells [14, 16, 18, 19]. Marked modulations in the distributional PG landscape of bone-marrow stromal cells and their associated ECM stand out as relay events in the control of the local engraftment, dissemination patterns and malignant progression of neoplastic hematopoietic cells.

## The proteoglycan control of hematopoietic stem/progenitor cell behavior

**Fig. 2:**
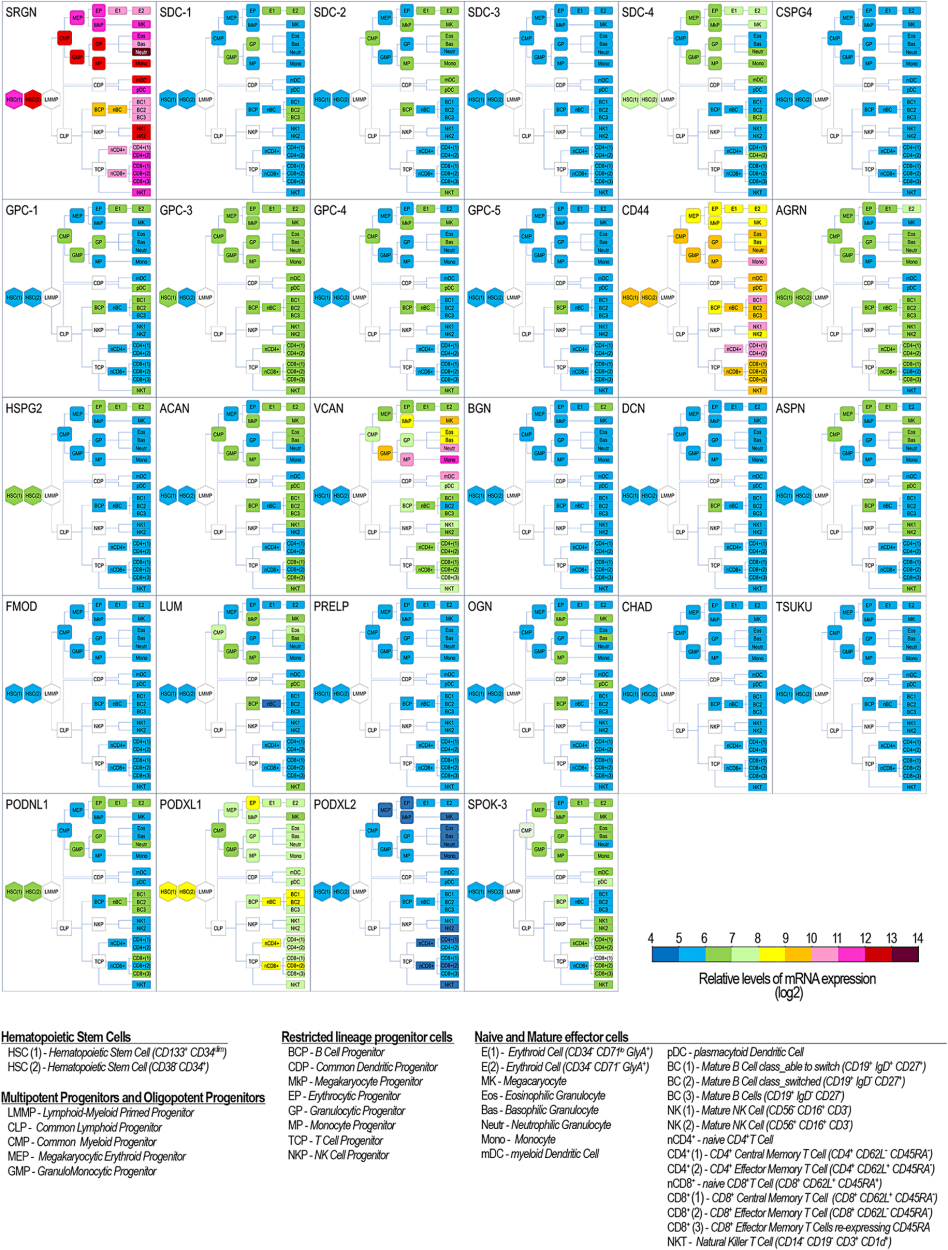
Schematic overview of the patterns of proteoglycan transcription (listed in Fig. 1) defined for the primary cell phenotypes of the healthy hematopoietic system and gathered through a re-elaboration of the combined information accessible through BloodSpot (Normal Human Hematopoiesis– DMAP– accessible at https://servers.binf.ku.dk/bloodspot/) and experimental data reported in the literature. Outlined phenotypic classification is according to the currently adopted one, revised in recent years to better portray the different components of the human hematopoietic system. Virtually no information is currently available about the patterns of proteoglycan expression in isolated common early myeloid and lymphoid progenitors, as well as in the more restricted megakaryocytic-erythrocytic and granulo-monocytic progenitors (*grey coding*)

**Fig. 3:**
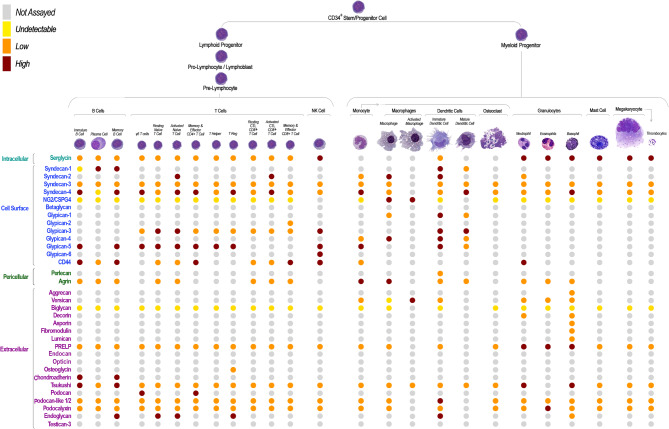
Schematic overview of the currently established patterns of proteoglycan core protein expression in the primary phenotypes of the healthy hematopoietic system. Proteoglycan expression patterns in stem and early progenitor cells remains to be better defined, whereas significant information is available regarding changes in proteoglycan synthesis occurring during their lineage progression

In the context of the hematopoietic system, *CD44* may be considered the “ancestral” cell surface-associated proteoglycan originally identified in its “full-length version” as an 85 kDa protein and denominated “*leukocyte-common antigen/lymphocyte homing receptor*“, or “*Hermes antigen*” [20–22]. Subsequently, it was established to represent an archetypal stem/progenitor cell membrane molecule [23]. Uniquely, *CD44* is one of the few PGs known to date to exhibit a ubiquitously high transcriptional rate and protein expression in both immature and terminally differentiated hematopoietic cells across lineages (Figs. 2–3). As a type I membrane-integral protein, endowed with a characterizing terminal Ig-loop domain and the ability to tightly associate with the cytoskeleton through the actin-binding ankyrin [24] and adaptor proteins of the FERM family [25, 26], the proteoglycan could be deduced to solely serve the purpose of acting as a transducing hematopoietic receptor for hyaluronan. However, decades of investigations have highlighted that *CD44* displays an array of molecular interactions that reach far beyond its hyaluronan-binding. It has been corroborated that the proteoglycan plays a multifaceted role in numerous hematopoiesis-associated cellular phenomena, including early specification of definite hematopoietic stem cells [27].

Much of the diversity in the *CD44*’s control of cellular events is attributed to the variation in its glycanation patterns, which are brought about by the alternative attachment to its core protein of heparan sulphates, different chondroitin sulphate isoforms or even dermatan sulphate, with in turn each of these GAG types undergoing intricate sulphation processes. Accordingly, it has been shown that *CD44* glycoforms carrying preferentially poorly sulphated chondroitin moieties activate monocytes, while, through a surface clustering mediated by binding to hyaluronan, the same variants facilitate the differentiation/maturation of the cells into macrophages [28]. The presence of highly sulphated chondroitin chains promotes lymphocyte activation, most characteristically that of B lymphocytes. In these cells, the B cell receptor/CD40-mediated activation induces upregulation of distinct *CD44* glycanation variants specifically substituted with heparan sulphates which are established to be key factors in the promotion of HGF signalling.

Molecular variants of *CD44* engineered such as to undergo alternative glycanation with either heparan or chondroitin sulphates has further shown that a hybrid LFA3-*CD44* molecule carrying chondroitin sulphates in the v3 exon-encoded domain specifically activates T lymphocytes through sequestration of RANTES [29]. Post-translational modifications unrelated to glycanation, but associated with defined sialo-fucosylations, create a selectin-binding glycoform of *CD44* denoted HCELL (“*Hematopoietic Cell E-selectin/L-selectin Ligand*”). This glycosylation variant is known to be abundantly expressed on hematopoietic stem/progenitor and dendritic cells and to act as an important adjunct for their transvascular trafficking dynamics [30]. Additionally, though its hyaluronan-binding activities, *CD44* may mediate T lymphocyte-dendritic cell interactions, believed to be fundamental for the triggering of immune responses, as well as for the promotion of the proliferation/activation of Th1^+^/CD4^+^ and Treg T-lymphocytes.

In germinal centres, *CD44* expression follows a bimodal trend during the course of B lymphocyte development (Figs. 2 and 3): high levels of expression of the proteoglycan are detected in CD23^+^/IgM^+^/IgD^+^ cells, while the proteoglycan is down-regulated in CD38^+^/CD23^−^/IgM^+^/IgD^−^ cells. Post-germinal centre IgG- or IgA-producing B lymphocytes re-express *CD44* to variable degrees [31], but to what extent the promoting role of *CD44* on B-cell maturation is connected to its hyaluronan-receptor function, or is a result of other molecular interplays, is not yet fully understood.

Another level of complexity of the *CD44*-mediated regulation of the hematopoietic progenitors’ development and the mature immune cells’ biology is afforded by the diverse actions ascribed to the alternative spliced isoforms of the proteoglycan. It was originally suggested that healthy peripheral blood lymphocytes primarily express *CD44* in its standard form, whereas restricted subpopulations of erythroid progenitors uniquely express the v10 isoform [31]. As a corollary, this specific isoform was documented to be critical in controlling hematopoietic stem cell engraftment and niche crosstalk [32]. Furthermore, *CD44* is frequently released from the cell surface by proteolytic cleavage, operated in both healthy and disease conditions [33–35], and its fragments may exert some of its matrix-associated functions at distance from the cell surface. The opposite, i.e., internalization of *CD44* followed by a cytoplasmic tail-mediated translocation into the nucleus, converts the proteoglycan into a putative transcription factor. Via formation of complexes with STAT3 and p300 [36], *CD44* is then capable of activating genes coding for cell-cycle regulators (e.g., cyclins and CDKs). Intriguingly, upon lymphokine stimulation of *CD44*-expressing NK cells (of the classical CD16^+^/CD56^+^ phenotype), the proteoglycan strongly contributes to the triggering of the cytotoxic activities exerted by these cells [37].

Conclusively, *CD44* remains a primary cell-membrane component sustaining the interaction of hematopoietic stem cells/progenitors with their niche environment. Through its combined interplays with cytokines and endogenously produced hyaluronan it is believed to be dispensable for correct differentiation of hematopoietic progenitor cells [38]. Coincidently, its hyaluronan-binding activity and close cooperation with the integrin α4β1 are widely recognized to be pivotal for the ensuing of intra- and extravasation phenomena and for the hematopoietic progenitors’ recirculation dynamics through primary and secondary lymphoid organs [39–43].

## Serglycin promotes cell differentiation within the myeloid lineage

Evidence has been provided suggesting that cytotoxic T lymphocytes and NK cells are highly dependent on *Serglycin* for the formation of their secretory granules [44], consistently with a primary function assigned to this proteoglycan in mast cells and their secretion-linked cell death pathways [45]. *Serglycin* is similarly present in the cytoplasm of differentiating neutrophils, eosinophils and megakaryocytes and is retained within the shed platelets ( [46–50]; Figs. 2 and 3). In these latter non-nucleated cells, it acts as a primary regulator of collagen-binding and cell-cell aggregation, thereby contributing to their overall thrombogenic efficiency. More specifically, platelet *Serglycin* is believed to assure proper packaging and secretion of selected α-granule proteins and the reduced secretion of dense granules [48, 49]. Because of the cell context- and immune event-dependent variations in its biological function, *Serglycin* has been defined as the “chameleon proteoglycan” of the immune system. Particular attention has recently also turned to the potential role of *Serglycin* in the regulation of the exosome biogenesis as a mean to impact on several cell biological phenomena. Combined bioinformatic and computational analyses of experimental data predict that *Serglycin* may interact with more than 40 different partner molecules including both intracellular and secreted factors and may have as a primary membrane interactor *CD44* [50]. This wide repertoire of putative molecular interactions characterizes the subcellular function of *Serglycin* and may ultimately explain its widespread abundance in a prevalent number of the leukemic variants, including those exhibiting an erythroid phenotype (see below).

## Contribution of cell surface-bound proteoglycans to the development of lymphoid lineages

To date, *CD44* is the proteoglycan to which an endothelium-transmigrating role is most solidly attributed. It is, however, axiomatic that other cell membrane-associated proteoglycans are equally vital for bone marrow-integration and the complex dissemination patterns undertaken by hematopoietic cell progenitors and their final derivatives. For instance, elective cooperation of *Glypican-3* with TFPI (“*Tissue Factor Pathway Inhibitor*”) promotes bone-marrow-homing and engraftment of transplanted hematopoietic stem cells, and this phenomenon cannot be reproduced by *Glypican-1* [51]. *Syndecans* expressed by B- and T-cell precursors are also postulated to participate in hematopoietic cell trafficking through their multivalent interactions with cell adhesion molecules, ECM components and motility-promoting factors. Although knowledge about the precise biological role of proteoglycans during hematopoiesis remains overall scanty, information has been gathered about the function of several proteoglycans in the control of differentiation/maturation and activation of selected phenotypes of the immune system. Unexpectedly, *Syndecan-2/CD362* has recently been found to be up-regulated in long-term renewing hematopoietic stem cells presenting a CD150^+^/CD48^−^/CD34^−^/c-Kit^+^/Sca-1^+^/Lineage^−^ phenotype and the proteoglycan has also been proposed to be directly implicated in their repopulation capacity through the control of p57 (Cdkn1c) and the cells’ cycling dynamics.

Patterning of proteoglycans is the most thoroughly documented through development of lymphoid lineages (Figs. 2 and 3). In fact, in a manner analogous to that observed for *CD44*,* Syndecan-1/CD138* glycoforms show an intriguing maturation-dependent bimodal expression pattern both throughout erythropoiesis [52] and during B lymphocyte development [53]. Discrete *Syndecan-1/CD138* variants are displayed on the surface of immature B-cell precursors, are rapidly disappearing at advanced stages of their maturation, and are reappearing in terminally differentiated plasma cells [53–55]. By contrast, *Syndecan-4* is constitutively expressed by early and late B-cell lineages [56] and is particularly enriched on activated CD4^+^ and CD8^+^ T lymphocytes, with a certain preference for effector CD45RA^+^ and CD45RA^−^ memory T-cells [57–59]. In general, primary B-cells express an ample repertoire of proteoglycans, which is strongly decimated upon modification of the phenotypic status of the cells induced in pathological conditions, as e.g., in the case EBV infection [60].

Rapid upregulation of *Syndecan-4* upon activation of CD4^+^ T cells is paralleled by p38 MAP kinase α/b-induced expression of *Syndecan-2* and both proteoglycans appear to be essential for T-cell activation [59]. Uniquely, activated T lymphocytes express an unusual glycanated variant of *Syndecan-4* capable of binding to an antigen-presenting cell membrane receptor known as “*dendritic cell heparan sulphate proteoglycan-dependent integrin ligand* (DC-HIL)”. Experimental evidence suggests that *Syndecan-4* synergizes the inhibitory function of this receptor through engagement of CD148 [58–61]. On the other hand, in their immature state, dendritic cells display a complex spectrum of membrane-bound proteoglycans including all *Syndecans* and *Glypicans-1*,* -3*,* -4* and − *5*. However, upon maturation they seem to down-regulate all these proteoglycans, except *Glypican-3*. Conceivably, many of the membrane-bound proteoglycans are essential for correct maturation of dendritic cells [62], since they facilitate and/or potentiate the cells’ responses to growth factors and signalling molecules, but, conversely, they may not be fundamental for their antigen-presenting functions.

Somewhat surprisingly, B and T lymphocytes, dendritic cells and macrophages also produce the ECM proteoglycan *Agrin*, known to be characteristic of basement membranes and more proper of the periosteal surface of the bone-marrow milieu. In T lymphocytes, *Agrin* appears to constitute a key element in their activation process [63]. However, the molecular mechanisms underlying this function remain veiled and to a certain extent far reached because of the peculiar tissue distribution of the proteoglycan. To note, however, is the finding that *Agrin* may control Eph-Eph receptor-signalling in the erythroid niche to propagate development of red blood cells by linking Eph receptor clustering to integrin activation [17].

## Dual role of cell surface-associated and ECM proteoglycans in the control of multiple myeloma and lymphoma progression

In multiple myeloma (MM) cells, the cytoplasmic proteoglycan *Serglycin* is uniquely secreted, rather than being retained intracellularly as in healthy hematopoietic cells and other diseased cells. Indeed, this is a remarkable condition given that in solid tumours several proteoglycans that are normally secreted or retained on the cell surface are frequently found within the cells. Through a chondroitin sulphate-mediated association with *CD44*, secreted MM *Serglycin* seems to be capable of exerting a direct control of cancer cell growth within the bone-marrow environment [64, 65]. More detailed analyses of plasma *Serglycin* detected in MM patients reveals that the proteoglycan acts a potent inhibitor of bone mineralization. This would suggest a dual role of the proteoglycan: [1] on one hand, it may directly interfere with bone formation (osteogenesis), while [2] it may modulate the balance between matrix deposition by osteoblasts and bone corrosion by osteoclast populations sustained by the myeloma cells.

**Fig. 4:**
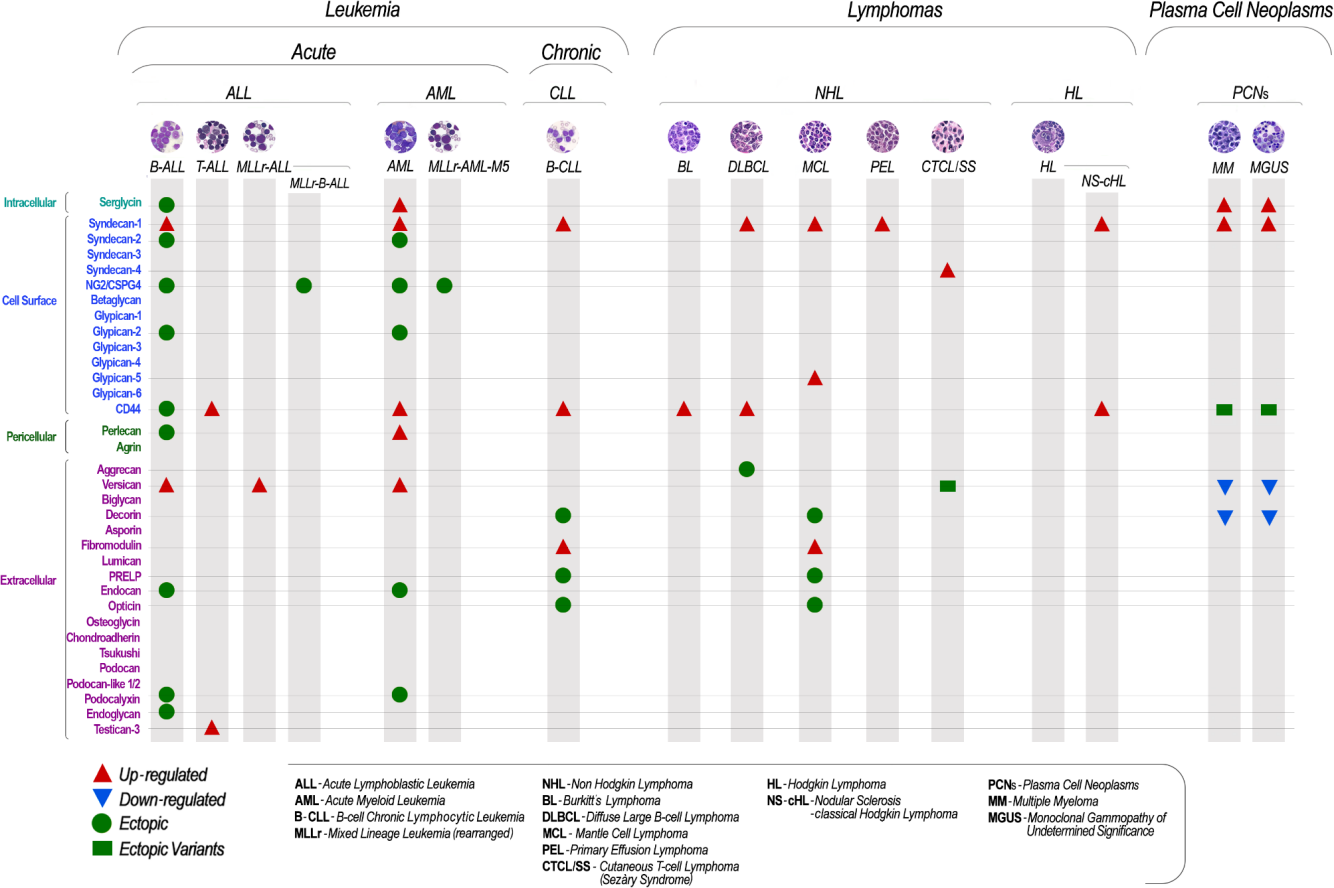
Schematic overview of the currently established expression patterns of proteoglycan core proteins in the primary classes of hematological malignancies

**Fig. 5:**
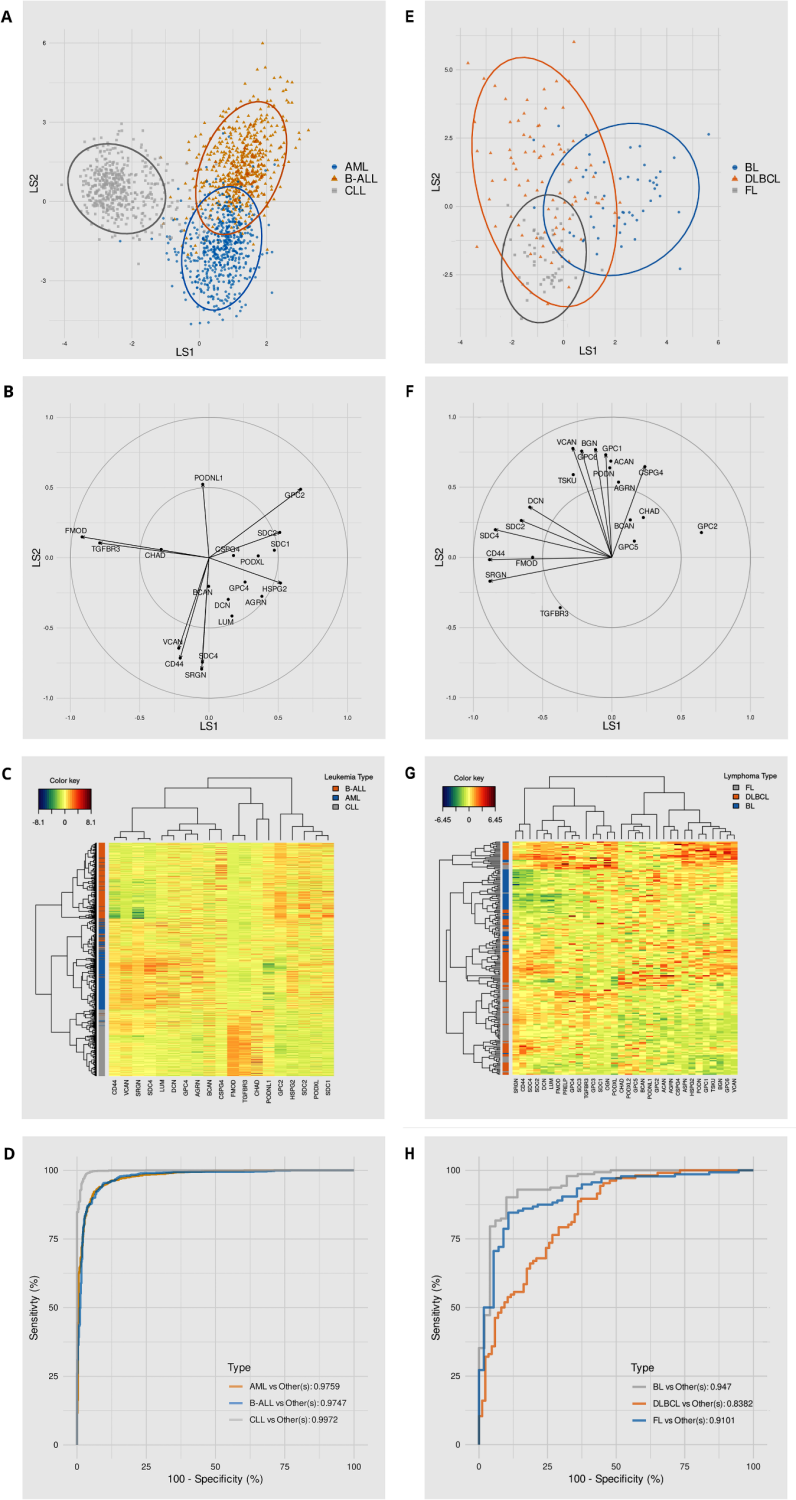
Potential discrimination of hematopoietic pathologies based on the transcriptome profiles of proteoglycans and their inter-relationship and computational analyses were founded on the model of Sparse Partial Least Squares-Discriminant Analysis (sPLS-DA). L*eft panel* refers to leukemia whereas *right panel* to lymphoma. Analyses were carried out by interrogation of publicly available transcriptional datasets and sPLS-DA models were trained with the aim to classify the three most represented leukemia subtypes, AML, B-ALL and CLL-B (**A**–**D**) and the lymphoma subtypes BL, DLBCL and FL (**E**–**H**). (**A**, **E**) Show latent structure projections for leukemia and lymphomas, respectively, where the coordinates of each sample are obtained by linear transformation of the relative expression of proteoglycan genes into a bidimensional (space) representations (LS1 and LS2), where the tumor variants are maximally discriminated. The figures show that samples of the same disease variant occupy specific areas of the plots, indicating that proteoglycan gene expression data has already enough information to discriminate the tumor variants. (**B**, **F**) The graphs illustrate the correlation of proteoglycan gene expression with latent structures as established by Pearson’s correlation coefficients of proteoglycan transcriptional rates with latent structure scores. Only genes included in the final schemes are reported and the 10 most influential proteoglycans are highlighted by arrows. For instance, the GPC2 gene in panel B points in the same direction of the group of B-ALL samples in panel A, indicating that GPC2 expression is more specific of B-ALL. Similarly, the FMOD gene is strongly correlated with CLL-B. (**C**, **G**) Expression heatmap composed by all proteoglycan genes that were used to build the models. The heatmaps and the two-ways clustering dendograms represented on the sides of the plots highlight the correlation between the expression of individual genes and specific tumor variant. For instance, FMOD and TGFBR3 are overexpressed in CLL-B (*panel C*). (**D**, **H**) One-vs-all ROC (*Receiver Operating Characteristics*). ROC curves allow to assess the model performance and indicate that, in particular for leukemia, the proteoglycan expression-derived models were effective in discriminating between tumor variants

Both T and B cell lymphomas display complex patterns of proteoglycan surface expression (Fig. 4), which are believed to be essential for supporting their cell-autonomous malignant behaviour and their microenvironmental interactions. Genome-wide analyses in lymphomas have evidenced recurrent high-level amplifications of proteoglycan genes as part of primary or secondary profiles of their genomic alterations. For instance, mantle cell lymphomas with the characteristic t(11;14)(q13;q32) translocation show frequent chromosomal gains involving *Glypican-5* [66, 67], albeit the tumorigenic implication of this gene amplification has not been further addressed. Extended proteoglycan transcriptional profiling in three primary lymphoma variants, i.e., follicular, Burkitt’s and Diffuse Large B Cell Lymphoma (DLBCL), highlights how these expression patterns differentiate the three lymphoma entities with > 80% specificity (Fig. 5E-H). Interestingly, transcriptional rates of proteoglycans preferentially bearing CS chains, versus those with HS chain glycanation, seem to be a discriminating factor.

The identification of *Syndecan-1/CD138* as a primary surface proteoglycan on MM cells prospected early on the potential pro-tumorigenic functions of upregulated surface proteoglycans (Fig. 4). It could be shown that MM *Syndecan-1/CD138* played an important role as an ancillary collagen type I co-receptor and as a modulator (mainly through its HS chains) of MM-bone-marrow interactions. Notably, this co-receptor function could be reproduced by engineering MM cell lines to ectopically express *Syndecan-2/CD362* or *Syndecan-4*, whereas it was not re-enacted by a similar manipulation of *Glypican-1* expression [68–71]. More recently, additional, more intricate roles have been attributed to MM *Syndecan-1/CD138* that stretch beyond the growth factor-binding, pro-angiogenic and pro-osteolytic properties of the proteoglycan [72, 73]. These include an important and more direct role for: [1] the process of growth factor-receptor activation; [2] the favouring of cell-cell communication between NK and MM cells, proposed to be operated via a surface clustering of extracellular histones; and [3] the protection of MM cells from treatment with next-generation proteasome inhibitors.

Since the early discoveries on the role of *Syndecan-1/CD138* on the MM cell biology, two fundamental notions have progressively been consolidated: *Syndecan-1/CD138* is indeed a reliable biomarker in MM, non-Hodgkin’s lymphomas, a number of plasmacytoid neoplasia and CLL-B and different isoforms of the proteoglycan are expressed in different B cell malignancies. One intriguing finding related to the expression of *Syndecan-1/CD138* on MM cells remains the fact that the discret cell subpopulations found to lack surface expression of the proteoglycan behave in a remarkably distinct way when compared to the majority of the MM cells with canonical overexpression of the proteoglycan. In fact, clonogenic *Syndecan-1/CD138*^−^ myeloma cells exhibit stemness traits and tumour-initiating properties that are normally associated with poorer prognosis. However, somewhat confounding, these cells may still show a characteristic plasma cell phenotype and may also be found in plasma cell leukemia patients [74–76]. These specific MM cell subsets have been reported to divide more infrequently and to be less invasive, due to their lack of expression of *Syndecan-1/CD138*. The tight correlation between *Syndecan-1/CD138* expression and aggressiveness of MM cells is corroborated by the enhanced intravasation and bone-infiltration capabilities exhibited by such cells in myeloma-bearing mice in which systemic treatment with an anti-*Syndecan-1/CD138* antibody interferes with dissemination of the cells [74].

In analogy to what has been observed in the context of the activation of healthy T lymphocytes, Sésary syndrome T cells overexpress *Syndecan-4* isoforms (Fig. 4) bearing discrete HS moieties capable of linking to DC-HIL and sequester TGFβ on the cell surface to suppress syngeneic and allogenic activation of T lymphocytes [60]. This would imply that these neoplastic lymphocytes may be equipped with multiple cell surface-bound proteoglycan species protecting them from both drug and immune system attacks. Surprisingly, although the upregulation of *Syndecan-4* detected in this rare T cell lymphoma may be in line with a transformation-dependent amplification of a constitutive transcriptional program, Sésary cells also aberrantly upregulate the *Versican* isoform V1 with ambivalent functional consequences. Secretion of the proteoglycan contributes to the enhanced migratory capabilities of the leukemic cells which are forged by an elevated skin tropism, while being sensitized to the action of chemotherapeutics [77]. To annotate in this context is the fact that Sésary syndrome cells ectopically produce the *Versican* isoform V1 and thereby secrete a matrix proteoglycan that is normally occupying their microenvironment of residency in the skin. The endogenously produced proteoglycan may therefore be utilized to promote their cutaneous infiltration capabilities and offer a growth support (and protection) at their ultimate localization sites. A further challenging finding is that ADAMST-mediated degradation of *Versican* causes a strong immunosuppressive condition in the microenvironment accommodating bone-marrow transplants. This observation strongly emphasises a pivotal role of the bone-marrow stromal ECM, assembled with a strong contribution of *Versican*, in the promotion of engraftment of transplanted hematopoietic stem cells.

The reversed scenario entailing down-regulation of matrix-associated proteoglycans rather than an up-regulation/de novo expression may also represent an impactful condition in haematological malignancies. Indeed, this is particularly noticeable in plasma cell malignancies (Fig. 4). This is for instance the case of ECM proteoglycans that may tightly associate with sequestration, latency and regulated activation of growth factors and/or signalling molecules. One of the most striking examples of such ability is that of *Decorin* in monoclonal gammapathies of undetermined significance (MGUS) and in MM, where reduced levels of *Decorin* enhance cell survival and spreading through a negative regulation of TGF and HGF signalling [78, 79]. In the bone-marrow microenvironment lodging malignant MM cells, *Decorin* deposition is contributed by the resident osteoblasts and seems to act as a direct antagonist to myeloma cell survival. In fact, experimental abrogation of *Decorin* through RNAi-based approaches, or the use of function-neutralizing antibodies, attenuates its survival-promoting effect on MM cells and their pro-angiogenic/pro-osteolytic effects. Conversely, increasing of the loco-regional levels of *Decorin* through forced expression in resident mesenchymal progenitor cells induces growth arrest of MM cells and the induction of programmed cell death.

Since it has been demonstrated that in different tissue districts (foremost cartilage) *Decorin* makes a significant contribution to the supramolecular assemblies and flexibility of “collagen-proteoglycan matrices”, it is conceivable that the modulatory role of *Decorin* in MM progression is balanced between its influences on growth factor-signalling dynamics and modifications of the architecture of the tumour microenvironmental ECM. The control of *Decorin* secretion in MM bone-marrow niches is dictated by the close interaction between the malignant plasma cells and immature osteoblasts and may be mediated by the cytokine CCL3. Intriguingly, current target-therapy drugs for standard-of-care treatment of MM that are directed against the proteasome seem to revert the suppressive effect of MM cells on *Decorin* production. Therefore, their wider application potential is further warranted by a potential anti-osteolytic action associated with the proteoglycan.

## Aberrant expression of cell surface-associated proteoglycans on leukemic cells tightly links to gene abnormalities, confers disseminative capabilities and contributes to the acquisition of drug resistance

In both childhood and adult acute T and B lymphoblastic (T-/B-ALL) and myeloid (AML) leukemia, complex and variant-specific patterns of proteoglycan expression are induced, resulting in de novo and/or enhanced transcription of both cell membrane-bound and secreted proteoglycans (Fig. 4). From a more in-depth analysis of the distribution/expression pattern of the single proteoglycans, *CD44* appears to be most ubiquitous proteoglycan across hematologic neoplasia, being also abundant on leukemia-initiating stem cells ( [32]; Fig. 4). Accordingly, it has consistently been observed to be upregulated in several leukemia types in which it seems to promote bone-marrow infiltration and increased tumour burden [31, 32, 80–84]. Malignant hematopoietic cells preferentially up-regulate *CD44* variants encompassing the v5-7 and v10 exons. This finding suggests that in hematopoietic malignancies, as in the case of numerous solid tumours, transformed cells upregulate the *CD44* splicing machinery to generate unique genetic variants of the proteoglycan with more articulated functions.

In experimental models of adult T-ALL and AML it has been possible to pinpoint *CD44* as a direct transcriptional target of Notch signalling, while other observations have implicated the proteoglycan as an inducer of the formation, persistence and progression of pre-leukemic and leukemic cancer stem cells [80, 83, 85, 86]. Interestingly, experimental data suggest that in AML patients acquiring resistance to the Bcl-2 inhibitor Venetoclax, the minimal residual disease detected in these individuals may be linked to a CXCR4 docking function of *CD44* upon CXCL12 engagement [85]. These and ancillary observations therefore suggest that overexpression of *CD44* may be considered a hallmark of leukemiagenesis and these findings incite the preclinical exploration of immunotherapeutic and CAR-T cell-targeting efficacy of *CD44* in diverse experimental leukemia models [32, 82].

Cytokine/RAS-induced *CD44* upregulation, or de novo transcription of the proteoglycan is not exclusive of B-ALL, but is also characteristic of T-ALL, AML [87, 88], myelodysplastic erythropoiesis, CLL-B [89] and more infrequent leukemia variants, such as erythroleukemia [90] and mast cell leukemia [91]. In all these leukemic conditions, immunological targeting of *CD44* (through direct antibody ligation) produces commonly denominating anti-tumour effects: a significant inhibition of cell proliferation by stabilization of the cyclin-dependent kinase inhibitors p21 and p27; down-regulation of cyclins and *c-jun*; and a concomitant triggering of PI3K/MAP/ERK-mediated survival-promoting signalling pathways sustained by the induction of caspase-independent calpain-elicited release of AIFs from mitochondria. This set of data lends support to the notion that signal transduction through extracellular engagement of *CD44* strongly influences the expansion of leukemic blasts. However, it remains to be established whether this effect is attributed to the canonical hyaluronan-binding receptor function of the proteoglycan, or whether it involves the eliciting of intricate signal transduction events mediated by cross-talking pathways triggered by other extracellular interactions of *CD44*.

Evidently, in both genetically construed mouse models of T-ALL and patient-derived T-ALL cells, enhanced expression of *CD44* confers chemoresistant properties, which are partly mediated by augmented drug effluxes [92]. In this case, however, binding of *CD44* to hyaluronan may be more convincingly implicated in the resistance mechanism since hyaluronidase treatment of the cells restores drug sensitivity. Particularly interesting also remains the role played by *CD44* in acute leukemia of myeloid derivation, as evidenced by the outcome of studies involving HOX10A-based transgenic mouse models and xenogenic transplantations. In such experimental paradigms of myeloid leukemiagenesis, *CD44* seems capable of purporting the activity of leukemia-initiating cells following their sequential transplantations into diverse hosts [4, 80, 82, 85, 93]. Thus, in the context of AML, mis-regulated *CD44* production may singularly assume proto-oncogenic functions. Transcriptional analyses and antibody-based protein detections performed on B-ALL and AML further identify the unparalleled concurrent expression of *Syndecan-1/CD138*, *Syndecan-2/CD362*, *Endoglycan*,* Podocalyxin-like 2* and *Glypican-2* (Fig. 4). However, the precise patterns of expression of these proteoglycans in diverse B-ALL variants and the pathological consequences of the aberrant transcription profiles are currently unknown.

MLL and KTMA2 gene aberrations characteristic of immature B-ALL and AML affecting both infants and adults strongly associate with de novo expression of *NG2/CSPG4* [4]. The marked prognostic impact of the de novo expression/upregulation of this surface proteoglycan in MLL rearranged adult and childhood leukemia has thoroughly been asserted by clinical correlation studies, which have led to the suggestion that expression of *NG2/CSPG4* may electively correlate with a stage-specific maturation arrest and neoplastic transformation. However, more recent studies have highlighted that AML cases manifesting a normal karyotype and FLT3 or NPM1 mutations may also exhibit an ectopic *NG2/CSPG4* expression. This finding together with the frequency of *NG2/CSPG4* expression on plasmacytoid dendritic leukemia cell lines lacking MLL abnormalities and displaying the immunophenotype CD34^+^/CD38^+^/CD45^+^/CD123^+^/HLADR^+^ have led investigators to assume that in the absence of MLL rearrangements, expression of the proteoglycan on leukemic cells may be dictated by the context within which leukemogenesis is initiated [94]. The consolidated correlations between gene rearrangements and unexpected *NG2/CSPG4* expression patterns still leave veiled whether ectopic transcription/translation of the proteoglycan is under a direct or indirect control of MLL gene rearrangements. However, a recent study has provided evidence that leukemic *NG2/CSPG4* is epigenetically controlled by such rearrangements, because of being transcriptionally regulated by the MLL-AF4 fusion protein [95]. Thus, enhanced *NG2/CSPG4* expression in leukemic cells may promote their aggressiveness in a cell-autonomous manner [95, 96] and thereby contribute to the ominous clinical course observed in such patients.

In MLL rearranged childhood pre-B ALL, enhanced surface expression of *NG2/CSPG4* is thought to potentiate the cells’ dissemination capabilities, including their ability to infiltrate the brain and hematopoietic sites [96]. Experiments combining patient-derived xenografting and immune-targeting of *NG2/CSPG4* strongly supports this idea and further highlight that immunotherapeutic blockade of *NG2/CSPG4* synergizes with standard-of-care drug treatment protocols featuring vincristine, gluococorticoids and L-asparaginase as primary agents. Interference of the *NG2/CSPG4* biological activity in these leukemic models prevents spreading of the neoplastic cells and induces complete remission and/or a significantly prolonged event-free survival of the transplanted mice [94]. Som of our unpublished observations that suggest that de novo/enhanced expression of leukemic *NG2/CSPG4* may be responsible for the cells’ resistance to chemotherapeutic drugs has recently been corroborated in the context of resistance to glucocorticoids by the observed direct interaction of *NG2/CSPG4* with FLT3 and the triggering of ligand-independent signaling and a concurrent downregulation of NCR3 [95]. The observed curative effect seen in animal models of leukemia growth following administration of anti-*NG2/CSPG4* antibodies has been attributed to a putative role of proteoglycan in agonizing with bone-marrow resident cells in creating a chemoprotective microenvironment. As bone-marrow mesenchymal stem/progenitor cells are widely known to express *NG2/CSPG4*, an extension of the above conjecture would be that antibody blockade of the proteoglycan may exert a bivalent destabilization of both the cancer cells and their microenvironmental supportive cells.

## Antithetic leukemogenesis-promoting effects of ECM-associated proteoglycans of the bone-marrow microenvironment

Computational analyses of the transcriptional profiles of an array of 32 proteoglycans, examined in AML and B-ALL and chronic lymphocytic B-cell leukemia (B-CLL), highlights that proteoglycan transcriptional rates may discriminate the three leukemia variants with a sensitivity reaching up to 90% (Fig. 5A-D). Interestingly, up-regulated transcription of *Fibromodulin* clearly segregates B-CLL from other B and T cell leukemia, whereas *Glypican-2* seems most exclusive of AML variants (Fig. 5B). It may also be noted that the up-regulation of *Versican* in tissue sites lodging B-ALL leukemic cell infiltrates is often accompanied by an ectopic expression of the proteoglycan by the leukemic cells themselves to further enrich the microenvironment with this proteoglycan and render it even more protective [97]. The AML variant characterized by mutation in the NMP1 (nucleophosmin) gene shows a frequent high expression of *Versican*, which is believed to be associated with the pathogenesis and clinical course of the disease [98]. Similar discoveries have been reported *Perlecan* [99] to exemplify a recurrent synergy between cell surface-associated proteoglycans and structural ECM-associated proteoglycans in promoting malignancy of hematopoietic neoplasia.

As a direct contrast to the tight correlation between overexpression of several proteoglycan types and a more aggressive phenotypic trait of leukemic cells, ATF3-regulated *Testican-3* (also carrying heparan sulphate chains) expression has been proposed to counteract T-ALL malignancy through impairment of MMP2 activity [100]. It remains uncertain to what extent loss of *Testican-3* expression may translate into a driving factor for diseased progression in T-ALL patients. Meanwhile, in AML patients, serum levels of another ECM proteoglycan, *Endocan*, show a disease- and therapy response-related variation [101, 102] and experimental knockdown of the proteoglycan in leukemic cells induces cell-cycle arrest and an NF-kβ-dependent activation of pro-apoptotic factors [103].

## Misregulated expression of small leucin-rich proteoglycans in leukemic cells

Some proteoglycans have emerged as up-regulated key genes in haematological malignancies within the framework of aberrant cancer-associated transcriptional programs (Fig. 5). One of the most remarkable and unexpected such case may be the one represented in CLL-B by the *de novo* transcription of *Fibromodulin* (a proteoglycan characterizing specialized connective tissues). Evidently, the atypical ectopic expression of the proteoglycan (that may reach 300-2,200 folds versus that of healthy lymphocytes) was initially discovered through differential gene screens and subsequently established to be a highly ranked gene aberration in CLL-B. The original discovery was further consolidated by numerous follow-up studies that extended the finding to mantle cell lymphomas, while confirming the absolute uniqueness of this proteoglycan expression in the two neoplasia [104]. Meanwhile, computational analyses have revealed that combined up-regulation of *Fibromodulin* and PI3K-C2B could unambiguously discriminate (i.e., with virtually 100% accuracy) between pre-leukemic polyclonal B cell populations/leukemic B cells and normal B lymphocytes [105].

As a remarkable corollary, in CLL-B and mantle-cell lymphomas de novo expression of *Fibromodulin* seems to be accompanied by a similar aberrant expression of *Decorin* [106] and two other small leucine-rich proteoglycans of the same subfamily, *Opticin* and *PRELP (prolargin)*. In addition, the latter two ECM proteoglycans exhibit a peculiar subcellular localization in the neoplastic lymphocytes being apparently confined to the endoplasmic reticulum and the nucleus [107]. Although the precise significance of the intracellular accumulation of these secreted proteoglycans remains to be defined, it may be envisioned that the proteoglycans concur with *Fibromodulin* in promoting enhanced CLL-B cell survival and drug resistance.

A direct tumorigenic function of *Fibromodulin* in CLL-B may indeed appear far reached, but several investigations have provided alluring hints on the oncogenic function of the proteoglycan. Aberrant translation of *Fibromodulin* in CLL-B cells seems to protect neoplastic lymphocytes from irradiation-induced apoptosis in a p53-dependent manner and, vice versa, RNAi-based knockdown of *Fibromodulin* in patient-derived CLL-B cells elicits regulated cell death [108]. Collectively these observations would then suggest that the proteoglycan operates in these leukemic cells as a survival-promoting factor. Although no additional data is available to substantiate these findings and/or provide clarifying mechanistic information, CLL-B cell-associated *Fibromodulin* has the potential to act as a potent promoter of the expansion of autologous tumour-specific T cells. This has been demonstrated in vitro when examining native CLL cells isolated from 13 different patients and pulsed with HLA-A2-binding *Fibromodulin* peptides [109]. The possibility is therefore prospected that specific *Fibromodulin*-derived peptides may be exploited as tumour vaccines for the treatment of CLL-B, unless it would turn out that such peptides may evoke collateral cartilage-directed inflammatory reactions.

## Prognostic valency of perturbed PG expression

**Fig. 6:**
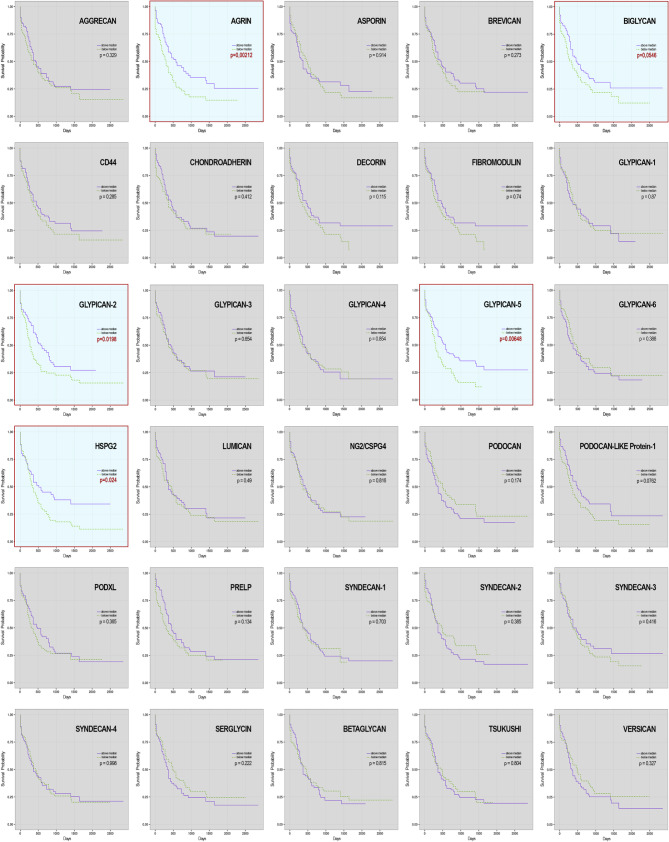
Survival probabilities in AML patients presenting aberrant transcriptional levels of the different proteoglycans. Kaplan-Meier curves are based on gene expression above or below median values determined for the given proteoglycan. Datasets were retrieved from The Cancer Genome Atlas (TCGA) https://www.cancer.gov/ccg/research/genome-sequencing/tcga and are based on: a combination of NGS-based singe-cell transcriptomics; the TCGA microarray platform (Affymetrix U133 Plus 2), including the MILE (Microarray Innovations in LEukemia) study on 2,096 patients; and BloodPool, an aggregated and integrated dataset grouping the results of multiple studies focusing on AML (see BloodSpot help and information page at https://servers.binf.ku.dk/bloodspot/)

Since altered expression of proteoglycans is invariably portrayed to be linked to the progression of haematological neoplasia and may in some cases be a contributing factor to their arising, it could be expected that aberrant expression patterns of several of the proteoglycans may be clinically meaningful (Fig. 6). Coherently, a clear prognostic impact has been ascribed to the upregulated expression of various proteoglycans in the neoplastic cells themselves, in their microenvironment, or as proteins detectable in sera of cancer patients. For instance, both upregulation of distinct splice forms of *CD44* (*CD44v6* and *CD44v9*) in the neoplastic cells and high levels of circulating *CD44* fragments typically correlate with overall survival, disease progression and recurrence in a spectrum of haematological neoplasia, including diffused large cell lymphomas (DLCL), plasma cell dyscrasias, i.e., MM, MGUS and myelodysplastic syndrome, Hodgkin’s disease and Burkitt’s lymphoma, B-CLL and childhood leukemia [34, 110–114]. As in the case of other proteoglycans, enhanced expression of *CD44* contributes to the malignant phenotype by affecting sensitivity of the neoplastic cells to chemotherapeutic drugs and the creation of resistant conditions [92, 115, 116]. These findings would prospect a dual benefit of an immunotherapeutic targeting of *CD44*, i.e., to both eradicate the neoplastic cells and revert pharmacological resistance of those cells failing to succumb the antibody action.

Much due to its decade-long history as a gold-standard surface marker to detect MM cells in the bone-marrow and in peripheral blood, *Syndecan-1/CD138* is an undiscussed prognostic factor in such tumour entity, when considered alone or in combination with CD38. In fact, selection of CD38^+^/CD138^+^ cells from MM patients enriches for a highly malignant population [117, 118], while cell surface-shedding of the proteoglycan and accumulation of *Syndecan-1/CD138* fragments in the bone-marrow tumour milieu and in circulation sets up a detrimental chemoresistance loop [119, 120]. ECM-associated proteoglycans may also be critically involved in the pathogenesis of MM as suggested by the fact that multivariate analyses indicate that reduced levels of *Decorin* are less favourable than high levels of the proteoglycan for optimal treatment responses of MM patients [121].

From a series of correlated investigations, it has been possible to convene that > 60% of leukemic patients harbouring MLL rearrangements ectopically express *NG2/CSPG4*, while when considering the reversed condition, upregulation of the PG coincides with such gene rearrangements in 80–100% of the examined leukemic cases (reviewed in ref. 4). Therefore, the consensus is that de novo expression of *NG2/CSPG4* tightly correlates with 11q23-linked genetic abnormalities and identifies a specific and well-defined subset of leukemic patients with dismal prognosis, as also exemplified by the outcome of the GMALL study on CD10^−^ adult pre-B ALL [4]. In fact, the originally proposed correlation between 11q23 genetic aberration and upregulated NG2/CSPG4 expression were subsequently further corroborated by computational and flow cytometric analyses performed on 1,461 infants with B-ALL. From this extended analysis it emerged that, although *NG2/CSPG4* expression seems to be restricted to a subgroup of patients genotyped with MLL rearrangements, virtually all patients presenting *NG2/CSPG4*-positive leukemic blasts harboured MLL rearrangements, i.e., *NG2/CSPG4* expression is asserted to be “diagnostic” of MLL gene aberrations.

Of apparently more disease-restricted prognostic valency are the serum accumulations of other proteoglycans in onco-hematological patients. Indeed, enhanced expression of *Serglycin*, *Syndecan-1/CD138*, *Perlecan*, *Endocan*,* Fibromodulin Podocalyxin* and *Versican* may be used in association with other biomarkers to further stratify children with ALL in lower and higher risk patients for receiving standard-of-care therapeutic treatments [104, 106, 122–125]. The same set of proteoglycans also serve as important serological indicators of the disease course in AML patients and may be exploited as prognostic factors independent of the mutational status in CLL-B patients [126]. In mature B-cell non-Hodgkin’s lymphoma they also represent important prognostic indicators and tightly correlate with disease recurrence and the therapeutic responses of the patients, including those evoked by bone-marrow transplantation. An intriguing observation in the context of the prognostic impact of proteoglycan misexpression is that miR144/199-induced down-regulation of bone-marrow stromal *Versican*, or its proteolytic processing, create an immunomodulatory GAG-free core protein fragment denoted “*versikine*” and establishes a prognostically favourable condition [127, 128]. Accordingly, assessment of bone-marrow *Versican* degradation has been adopted as an important parameter in a Phase II trial evaluating the outcome of an autologous dendritic cell/myeloma cell fusion vaccination protocol (trial number NCT02728102).

Conclusively, since a prognostic value of several other proteoglycans has been demonstrated by both mRNA and protein measurements for a myriad of hematologic pathologies, when considering virtually all clinical parameters/conditions, i.e., overall survival, disease-free survival, minimal residual disease and therapeutic response, including beneficial effect of autologous and allogenic bone-marrow transplantation. Accordingly, publicly accessible transcriptome analyses on roughly 300 non-randomized patients affected by AML (considering all variants) highlight that upregulation/de novo transcription of a least five distinct proteoglycans, including *Agrin*, *Biglycan*, *Perlecan*, *Glypican-2*, *Glypican-5* and *Podocan-like protein-1*, significantly impacts on overall survival of the patients over a 9/10-year follow-up period (Fig. 6). It can be envisioned that strengthening of the statistical power and reproducibility of the current findings through enlarged multicenter studies is predicted to reinforce the prognostic significance of perturbed proteoglycan expression throughout the onco-hematologic landscape.

## Prospective clinical targeting of proteoglycans

Aberrantly expressed proteoglycans are natural targets for both active and passive immunotherapeutic approaches, albeit, remarkably, their therapeutic exploitation has not significantly progressed into conclusive clinical experimentation and/or to a due number of explorative trials. The reason for the lack of a more determining impact of proteoglycans as effective immunotherapeutic targets is currently not understood and the suspicion remains that they may have been overlooked. For instance, effectiveness of immunotargeting NG2/CSPG4 in solid tumours, both “passively” and “actively” (with a wealth of agents and approaches), has been appreciated and therefore transposition of these targeting approaches to B-ALL and discrete AML variants would seem implicit. Analogously, the precedent targeting of *Glypican-3* in hepatocarcinoma should spur a greater attention to the potential of *Glypican-2* and *− 5* in several hematologic tumours.

The advantage of opting for proteoglycans as immunotargets is strongly sustained by the unparalleled opportunity to exploit antibodies recognized cancer-selective glycoforms of the macromolecules, such as to avoid collateral targeting of more ubiquitous variants widely expressed in human tissues. To date, some information about the clinical potential of proteoglycan for cancer treatment may be gained from the few first-in-man dosing efforts that have been performed. For instance, mAbs inhibiting *CD44* binding to hyaluronan have stood up as candidate immunotherapeutic agents for treatment of B-CLL [129, 130], much because of the tight signal transduction linkage of CD44 with ZAP70 (a recognized prognostic CLL-B marker). In this context, the advent of the CAR-T technology has bolstered the exploitation of cancer-targeting immunological (antibody-based) and non-immunological (mRNA-based) approaches, such as those adopted in the case of the pilot, open label multicentre Phase 1/2a study of the MLM-CAR44.1 T-cell agent. It was pursued on AML and multiple myeloma patients (NCT04097301; [131]), but due to its early termination, a comprehensive evaluation of the outcome could not be accomplished. A more recently initiated Phase 2 trial (NCT02046928), promoted by Ångstrom Pharmaceuticals, features the use of a CD44-binding peptide (coded “A6”) for the treatment of CCL-B. Conceivably, a caveat when attempting passive or active cancer eradication through CD44 may turn out to be its widespread distribution in healthy tissue of the body. This drawback could be circumvented by the development t of anti-neoplastic antibodies specifically recognizing discrete cancer-elective splice variants of the proteoglycan.

Immunotargeting of *Syndecan-1/CD138* appears to be an appropriate and putatively successful approach for MM treatment, given the wealth of supportive experimental data and convincing preclinical results obtained by radioimmuno-targeting through a ^213^Bi-antibody conjugate [132]. Accordingly, the fully humanized anti-*Syndecan-1/CD138* ADC Indatuximab ravtansine (nBT062; maytansinoids as drug load) has shown highly promising preclinical results in vitro and in vivo, especially when applied in combination with standard-of-care targeted drug treatments [133, 134]. Hence, a cogent multicentre clinical Phase 1/2a study has been carried out to explore the efficacy of Indatuximab lenalidomide and the outcome of this trial provided a clear indication of a well-tolerated anti-tumour activity of the agent on a cohort of 64 patients affected by refractory MM [135]. There are therefore high expectations on the outcome of the second phase of the study. A CAR-T cell targeting approach has also been contemplated for *Syndecan-1/CD138* and this has been done through the ATLCAR.CD138 cells applied to MM patients.

The ongoing interventional Phase 1 study (initiated in 2019) entails administration to relapsed and/or refractory MM patients of autologous CAR-T cells targeting the *Syndecan-1/CD138* antigen. Due to the exceptionally long follow-up (15 years) conventionally adopted by the International Myeloma Working Group for the evaluation of overall survival, progression-free survival and overall response rate, no information is yet available about the outcome of this trial. In summary, emerging CAR-T cell therapeutic approaches may become the forefront of the exploitation of proteoglycan antigens, as firmly suggested by the promising preclinical efforts with CAR-T-based approaches targeting *CSPG4* and *Syndecan-1/CD138* in childhood and adult acute leukemia. However, advancement of next-generation ADC compounds and engineered antibody platforms for active immunotherapy may ultimately propel these targets to wider clinical applications.

## Conclusions

**Fig. 7:**
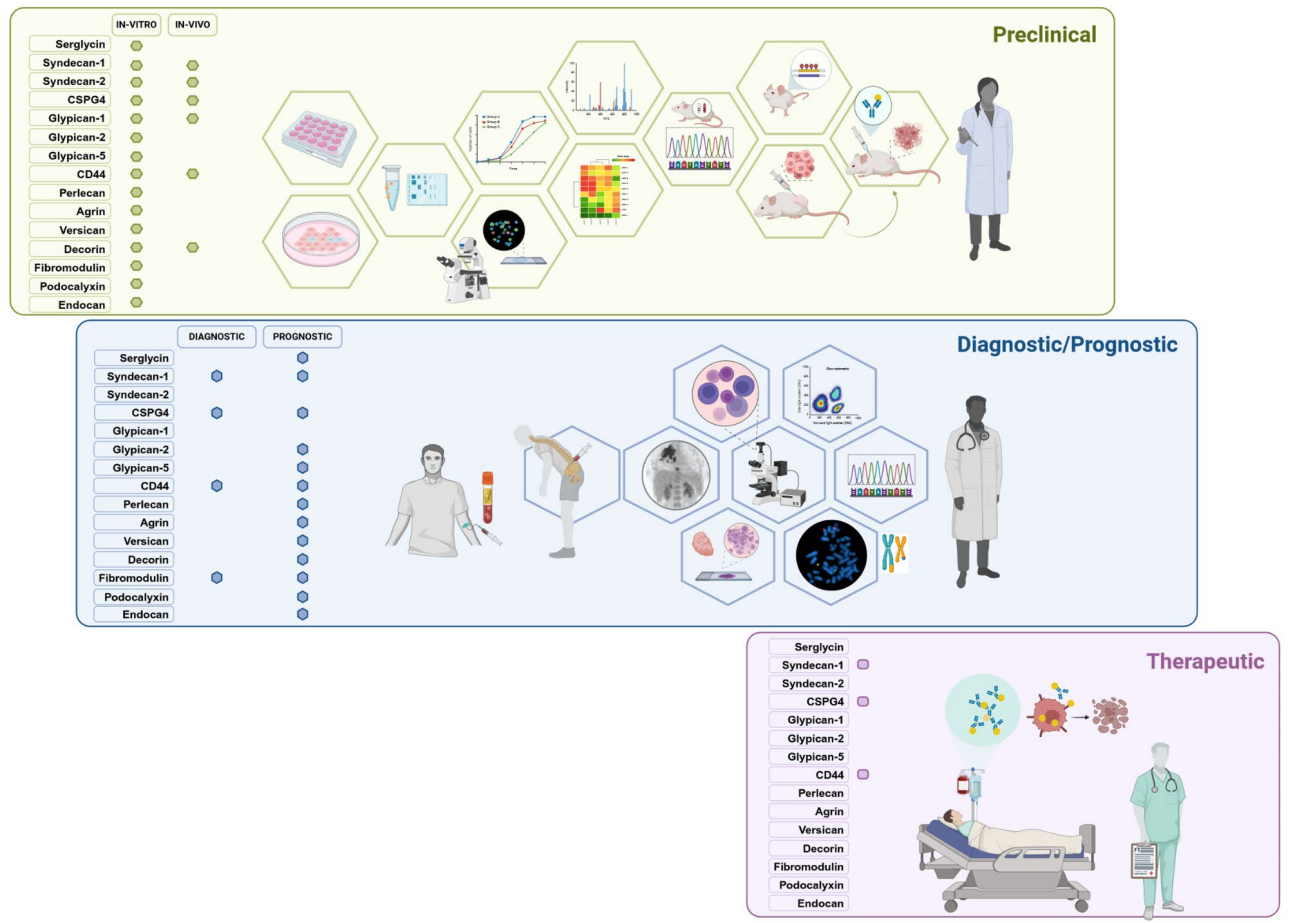
Overview of the currently outlined clinical potentials of proteoglycans as tumor and therapeutic targets. Comprehensively, diagnostic/prognostic significances are attributable to alteration in the proteoglycan surface expression that are disclosable on neoplastic hematopoietic cells; to perturbed expression of proteoglycans in their microenvironment; and/or to aberrant accumulation of the intact or fragmented molecules in the blood of cancer patients. Decades worth of experimental work in vitro and in vivo has clarified some aspects of the biological role of proteoglycans in hematopoietic malignancies and consolidated their valency as putative therapeutic targets. To effectively implement the clinical exploitation of these targets a major leap would need to be taken in the actual transfer of the findings from preclinical developmental phases to more advanced clinical experimentation. It is likely that the undertaking of such leap has been hampered by the lack of adequate reagents for targeting of cancer-specific glycoforms and/or the effective exploitation of next-generation therapeutic platforms, while the actual clinical potential of some proteoglycan may have failed to be fully appreciated

Proteoglycans are recognized multivalent regulators of the development and renewal of the hematopoietic system while, upon neoplastic transformation, their expression is frequently altered to purport a myriad of molecular interactions and drive pivotal processes taking place within the cancer cells and their microenvironment. Predictably, one or more proteoglycans are directly or indirectly involved in the control of each of the established cancer hallmarks, inciting a closer attention to be paid to the defining of their precise expression patterns and the unfolding of the array of functions they may exert in neoplastic conditions. In such conditions, currently accrued data support an effective transfer of laboratory data to putative diagnostic and therapeutic approaches targeting proteoglycans (Fig. 7). Undeniably many of these macromolecules, and especially the cell surface-bound ones, are fully druggable and can readily serve as immunotherapeutic targets. Aberrant expressions of proteoglycan may therefore deserve a more accurate evaluation to determine their significance for the progression of hematopoietic tumours and for the design of antibody- and cell-based anti-neoplastic therapeutic modalities. To implement all this there is a need to better understand the nature of the hematopoietic *Proteoglycome* that is represented in the healthy and diseased hematopoietic system and how we can take advantage of such knowledge to ameliorate our efforts to treat onco-hematologic conditions. This essay may constitute the basis for making such advancements.

## Data Availability

The datasets analysed for normal hematopoiesis are available in the BloodSpot - Normal Human Hematopoiesis (DMAP) repository at https://servers.binf.ku.dk/bloodspot/. The datasets analysed for leukemia are available at the https://ftp.ncbi.nlm.nih.gov/geo/series/GSE13nnn/GSE13159/matrix/GSE13159_series_matrix.txt.gz repository (htttp://10.1038/leu.2010.22). The datasets analyzed for lymphoma are available at the https://cbioportal-datahub.s3.amazonaws.com/mbn_mdacc_2013.tar.gz repository (10.3324/haematol.2020.274258).

## Abbreviations

ADC: Antibody Drug Conjugate
AIF: Apoptosis inducing factor
B: ALL–B–cell Acute Lymphoblastic Leukemia
T: ALL–T–cell Acute Lymphoblastic Leukemia
AML: Acute Myeloid Leukemia
CAR: T–Chimeric Antigen Receptor–T (cells)
CLL: B–B–cell Chronic Lymphocytic Leukemia
CSPG4: Chondroitin Sulfate Proteoglycan 4
DC: HIL–Dendritic Cell Heparan sulphate proteoglycan–dependent Integrin Ligand
DLCL: Diffused Large Cell Lymphomas
MGUS: Monoclonal Gammapathies of Undetermined Significance
MM: Multiple myeloma
NG2: Neural–Glial antigen 2
NMP1: Nucleophosmin
TFPI: Tissue Factor Pathway Inhibitor
